# Ustekinumab Exposure in Pregnant Women From Inflammatory Bowel Disease Clinical Trials: Pregnancy Outcomes Through Up To 5 Years in Crohn’s Disease and 2 Years in Ulcerative Colitis

**DOI:** 10.1093/crocol/otac025

**Published:** 2022-07-03

**Authors:** Bincy P Abraham, Elyssa Ott, Christopher Busse, Conor Murphy, Lindsay Miller, Daniel C Baumgart, Ellen Scherl, Christopher Gasink

**Affiliations:** Department of Medicine, Houston Methodist Academic Institute, Houston, Texas, USA; Department of Medicine, Weill Cornell Medicine, New York, New York, USA; Medical Affairs, Janssen Scientific Affairs, LLC, Horsham, Pennsylvania, USA; Medical Affairs, Janssen Scientific Affairs, LLC, Horsham, Pennsylvania, USA; Immunology, Janssen Research & Development LLC, Spring House, Pennsylvania, USA; Medical Affairs, Janssen Scientific Affairs, LLC, Horsham, Pennsylvania, USA; Division of Gastroenterology, University of Alberta, Edmonton, Alberta, Canada; Department of Medicine, Weill Cornell Medicine, New York, New York, USA; Medical Affairs, Janssen Scientific Affairs, LLC, Horsham, Pennsylvania, USA

**Keywords:** ustekinumab, pregnancy, Crohn’s disease, ulcerative colitis, inflammatory bowel disease

## Abstract

**Background:**

While no adverse developmental outcomes were observed in preclinical animal studies, limited data exist regarding effects of ustekinumab on human pregnancies. Previously, no data have been reported for women treated with ustekinumab in inflammatory bowel disease (IBD) clinical trials and corresponding pregnancy outcomes. Here, we present pregnancy outcomes from IBD clinical trials, incorporating 5 years of treatment in Crohn’s disease (CD) and 2 in ulcerative colitis (UC).

**Methods:**

All patients in the clinical trials agreed to use adequate birth control and were discontinued from treatment upon pregnancy confirmation. Nonetheless, 39 pregnancies occurred with maternal ustekinumab exposure from 4 CD and 1 UC study. Maternal and neonatal outcomes and data are presented with summary statistics, where available.

**Results:**

Of 1289 women who received ≥1 dose of ustekinumab, 39 maternal pregnancies with outcomes were reported (pregnancy cohort). Median maternal age was 28.0 years and median duration of ustekinumab treatment before pregnancy was 63.7 weeks with the last dose of ustekinumab administered prior to or during the first trimester (terminal half-life of ~3 weeks). Outcomes for the 39 pregnancies were: 26 live births (all normal newborns), 8 spontaneous abortions, and 5 elective abortions. No congenital anomalies were reported among normal newborns and no safety signals emerged with neonatal outcomes.

**Conclusions:**

Based on this series of 39 pregnancies with outcomes from IBD clinical trials, mothers treated with ustekinumab (limited to up to the first trimester) did not demonstrate a risk of negative outcomes. More data are needed to characterize the safety profile of ustekinumab use during pregnancy.

## Graphical Abstract

**Graphical Abstract:**
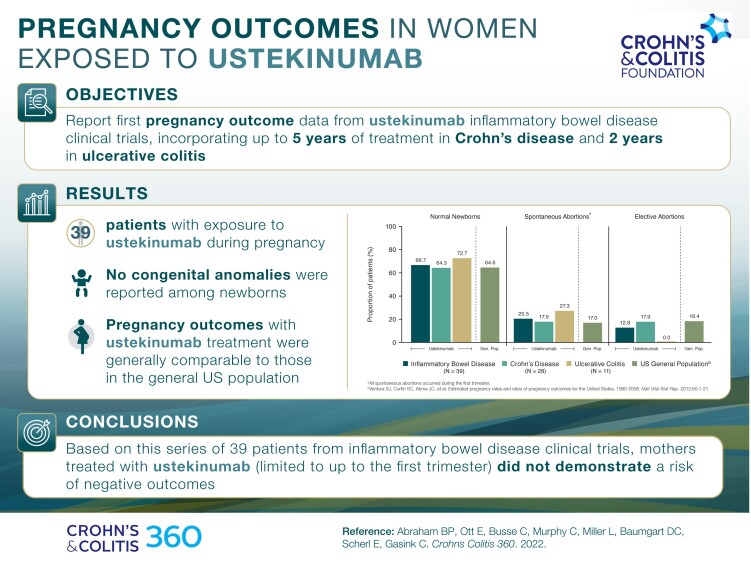

## Introduction

Crohn’s disease (CD) is a chronic inflammatory bowel disease (IBD) with complex diagnosis involving history and physical findings,^1^ while ulcerative colitis (UC) is a chronic inflammatory disease of the large intestine,^2,3^ but in both diseases many diagnosed patients are of child-bearing age.^4^ Ustekinumab is a fully human monoclonal antibody to interleukin 12/23p40 and was first approved for IBD in the United States initially for CD in 2016, and then for UC in 2019.^5^ Limited published data exist concerning the effects of ustekinumab and other more recently approved therapeutics on human pregnancies in patients with immune-mediated diseases.^6–8^

Ustekinumab has been shown to safely induce and maintain clinical response and remission through 1 year and long term, with up to 5 years of data in CD and 2 years in UC.^9–13^ The approved ustekinumab dosing in adults in the United States with CD and UC, is a single, weight-based intravenous infusion (approximating 6 mg kg^−1^), followed by 90 mg subcutaneous (SC) injection doses every 8 weeks (q8w).^14^ An additional maintenance dose of 90 mg SC every 12 weeks (q12w) is also approved outside the United States in the European Union and other regions.

No adverse developmental outcomes (prenatal or postnatal) were observed in preclinical (animal) studies of ustekinumab where serum ustekinumab concentrations in pregnant monkeys were >100× the serum concentrations in human patients treated subcutaneously with 90 mg ustekinumab weekly for 4 weeks.^14^ Across clinical trials in approved indications, 6710 patients have received at least 1 dose of ustekinumab treatment with approximately 14 000 patient-years of follow-up. In IBD clinical trials, 2575 patients were treated with ustekinumab with 3960 patient-years of follow-up.

The outcome of pregnancy in patients with CD or UC is impacted by the disease state throughout pregnancy, from conception to early and late pregnancy.^15^ Guidelines from the American Gastroenterological Association IBD Parenthood Project recommend treatment with biologics be continued throughout pregnancy given the known risk that poorly controlled disease can have on pregnancy outcomes.^16^ A study based on data from the Organization of Teratology Information Specialists (OTIS) Research Center at the University of California San Diego noted an association between maternal disease, specifically UC, and infant hemangiomas, with no significant difference noted among mothers treated with a biologic during pregnancy.^17^ In treating women with IBD, data related to pregnancy outcomes with biologic use during pregnancy, even when limited to small cohorts, is an important component of treatment selection.

Registry studies, such as TREAT^18^ and PIANO,^19^ along with real-world evidence, play an important role in pregnancy outcome data generation. The OTIS Research Center at the University of California San Diego is one such specialty organization providing observation pregnancy outcome data on exposure during pregnancy and dysmorphic infant evaluations. Meta-analyses are also useful in reporting the benefit–risk of biologic treatment in pregnancy for women with immune-mediated diseases.

A previous report of 13 years of data from the TREAT Registry showed that infants born to patients with CD who were treated with infliximab during gestation had similar outcomes to those without infliximab exposure.^18^ As noted in the TREAT report and additional publications, a lower live birth rate was observed in women with CD,^19^ although this may be attributed to a population with more severe disease and additional exposure to immunosuppressants. Results from the PIANO registry support biologic treatment during pregnancy in women with IBD as treatment exposure was not found to be associated with increased maternal or newborn adverse events (AEs).^20^ In a prospective cohort study from OTIS, no evidence of an increased risk of major structural birth defects was found in patients with CD who were exposed to adalimumab (11.1%) vs those not exposed to adalimumab (8.8%) as well as a wide range of other pregnancy outcomes attributable to prenatal exposure to adalimumab.^21^ Additionally, for women being treated for CD, this study confirms previous findings regarding elevated risks for preterm delivery associated with active maternal disease and rates of major congenital anomalies were higher than the 2%–4% reported in the general population.^14^

In a meta-analysis, a higher risk of preterm birth and spontaneous abortion, compared with the general population, was observed with the use of tumor necrosis factor (TNF) antagonists, although results from other studies have found that this risk is comparable to non-TNF antagonist users with immune-mediated disease. In a study of pregnant women with chronic inflammatory disease, the risk of congenital anomalies was not found to be increased with TNF antagonist exposure during pregnancy.^22^

Here, we report the first pregnancy outcome data from ustekinumab IBD clinical trials, incorporating up to 5 years of treatment in CD and up to 2 years in UC, including information about maternal disease activity and quality of life. This will add to the currently available data regarding treatment with biologics, specifically ustekinumab, during pregnancy in women with IBD.

## Materials and Methods

### Analysis

All reported maternal pregnancy cases with an associated outcome in IBD clinical trials are included in this analysis. Data are summarized and presented as frequencies and summary statistics, as appropriate.

### Patient Population

**Figure 1.**
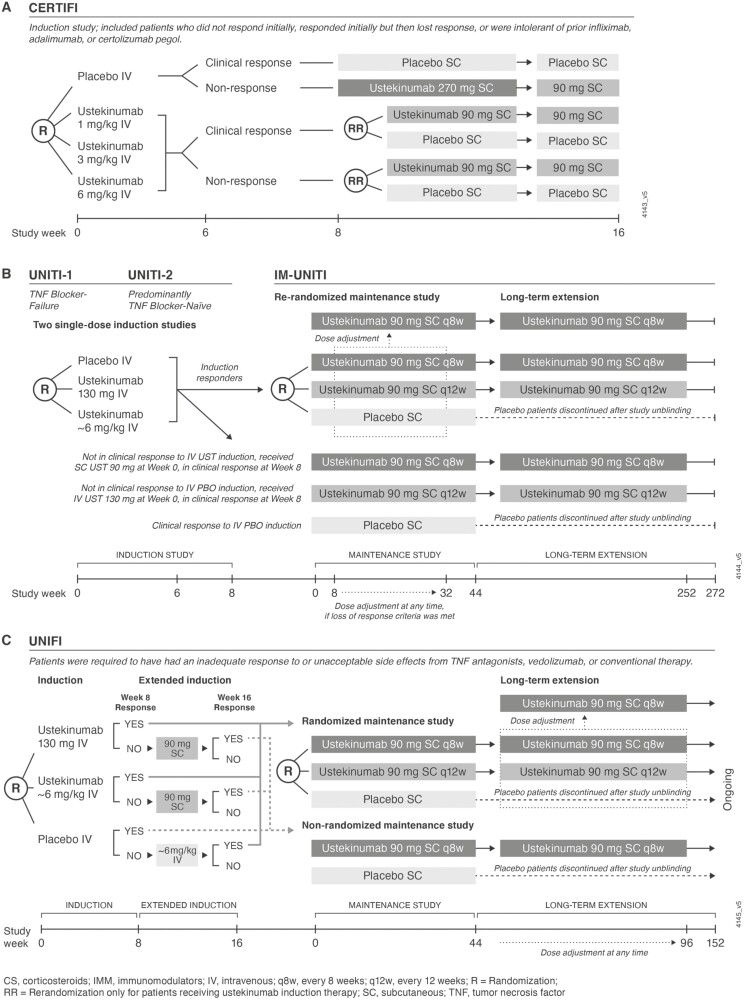
Details of the included Crohn’s disease (A, B) and ulcerative colitis (C) studies.

Study designs and corresponding descriptions of patient populations in the clinical trials have been previously reported for the Phase 2 and Phase 3 studies in patients with CD^9,23,24^ and for 1 Phase 3 study in patients with UC.^10^ Most studies included an IV induction phase and an SC maintenance phase with ustekinumab treatment q8w or q12w. Additional details of the 4 Phase 2 and Phase 3 CD studies (CERTIFI, UNITI-1, UNITI-2, and IM-UNITI) and 1 Phase 3 UC study (UNIFI) are provided in Figure 1. All patients had agreed to use adequate birth control, per protocol, and were discontinued from further study treatment once pregnancy was confirmed. Patients were followed for safety through 20 weeks after the last dose of study treatment. In addition, follow-up information was requested regarding the pregnancy outcome for both patient and infant. No maternal pregnancies were reported in Phase 2 study CNTO 1275T07;^23^ therefore, it is not included in this descriptive summary.

Enrollment in the CD and UC studies was limited to patients ≥18 years of age who had had either CD or UC for ≥3 months that was moderate to severe (with a score for the CD Activity Index [CDAI] of 220–450^25^ (out of a possible range of 0–600) or a total score of 6–12 on the UC Mayo scale (out of a possible range of 0–12^26^) and a subscore of 2 or 3 on the endoscopic component of the Mayo scale), respectively.

### Data Collection and Safety Outcomes

For all pregnancies, the following maternal information was requested: age, disease duration, previous pregnancies, ustekinumab use (dose, route, and frequency), medical history, smoking history, and concomitant medications. Additionally, systematically collected disease-related data included: CDAI (CD)/Mayo score (UC), health-related outcomes (36-Item Short Form Health Survey [SF-36]^27^ and the Inflammatory Bowel Disease Questionnaire [IBDQ]^28^) at baseline and before pregnancy. For IBDQ, a mean increase of ≥16 points from baseline is considered clinically meaningful and for SF-36, a mean change from baseline of ≥5 points; US general population norm is 50 ± 10.

Pregnancy outcomes were collected as either live birth (normal newborn or congenital anomaly with details collected as appropriate), elective abortion, spontaneous abortion, or stillbirth. Information and data on the infant were requested, including delivery method, sex, weight, length, Apgar score, and any additional medical therapy required.

### Ethical Considerations

Institutional review boards approved all study protocols and all patients provided written informed consent.

## Results

### Patient Demographics and Characteristics at Baseline

Overall, 39 maternal pregnancies with an associated outcome were reported out of a total of 1289 women randomized into IBD clinical trials; 28 in CD and 11 in UC. Of these, in patients with CD, 1 case is from the Phase 2 CERTIFI study, and the remainder are from the Phase 3 trials with 18 pregnancies reported in the UNITI-1/IM-UNITI study (TNF antagonist-failure population) and 9 in UNITI-2/IM-UNITI (conventional therapy failure population). All 11 cases in UC were from the UNIFI Phase 3 trial, including 2 pregnancies in TNF antagonist-failure patients, 2 in TNF- and vedolizumab-failure patients and 7 pregnancies in nonbiologic failure patients. In all cases, mothers received their last dose of active study agent, ustekinumab, prior to or during the first trimester (half-life of ustekinumab is approximately 3 weeks^14^).

Baseline characteristics for the overall IBD population, overall female only population, and the IBD pregnancy cohort are presented in Table 1. Patients in the pregnancy cohort had a lower median age than female patients overall (28.0 pregnancy cohort vs 39.0 female population). These female patients had similar median baseline CDAI scores in CD (300.5 pregnancy cohort vs 310.0 female population) and baseline Mayo scores in UC (6.0 pregnancy cohort vs 6.0 female population).

**Table 1.** Baseline characteristics.

	IBD population^a^	Female population	Pregnancy cohort
*N*	2529	1289	39
Age, median years (range)	38.0 (18, 84)	39.0 (18, 84)	28.0 (18, 42)
Race			
White	2105 (83.2)	1110 (86.1)	34 (87.2)
Women, *n* (%)	1289 (51.0)	1289 (100)	39 (100)
Child-bearing age (18–44 years), *n* (%)	841 (33.3)	841 (65.2)	39 (100)
Smoker (past or current)	451 (17.8)	268 (20.8)	10 (25.6)
Relevant medical history, *n* (%)^b^			
*N*	1701	962	28
Diabetes mellitus	52 (3.1)	34 (3.5)	0
Hypertension	228 (13.4)	128 (13.3)	1 (3.6)
Hyperlipidemia	99 (5.8)	59 (6.1)	0

Abbreviation: IBD, inflammatory bowel disease.

CERTIFI, UNITI-1, UNITI-2, IM-UNITI, and UNIFI.

Collected in Crohn’s disease studies only.

Across all female patients, a higher percentage in the pregnancy cohort reported current smoking (25.6% pregnancy cohort vs 20.8% female population; Table 1). The pregnancy cohort reported only 1 patient with a history of hypertension and no patients with a history of diabetes, or hyperlipidemia (relevant medical history only collected in CD studies; Table 1).

For all 39 pregnancies in the IBD pregnancy cohort, the median disease duration was 6.8 years (Table 2). The median duration of ustekinumab exposure prior to the reported pregnancy was 63.7 weeks and all patients discontinued ustekinumab on or prior to the reported onset date of pregnancy. More than half of all patients in the IBD pregnancy cohort (59.0%) had previously failed at least 1 TNF antagonist.

**Table 2.** Baseline demographics and disease characteristics for maternal pregnancies by neonatal outcome in the IBD pregnancy cohort.

	Neonatal outcomes in UST-treated IBD pregnancy cohort			
	Live birth	Spontaneous abortion	Elective abortion	Overall
*N*	26	8	5	39
Median maternal age, years (range)	27.0 (20, 35)	29.5 (22, 33)	27.0 (18, 42)	28.0 (18, 42)
Median duration of disease, years (range)	7.1 (0.3, 20.9)	5.1 (0.8, 12.7)	6.8 (1.9, 20.6)	6.8 (0.3, 20.9)
Median duration of ustekinumab treatment, weeks (range)	72.3 (0.1, 232.9)	76.4 (16.7, 121.1)	8.3 (0.1, 80.1)	63.7 (0.1, 232.3)
Tumor necrosis factor antagonist failures, *n* (%)	12 (46.2)	6 (75.0)	5 (100.0)	23 (59.0)
Current smoker, *n* (%)	7 (26.9)	2 (25.0)	1 (20.0)	10 (25.6)

Abbreviations: IBD, inflammatory bowel disease; UST, ustekinumab.

### Outcomes

**Figure 2.**
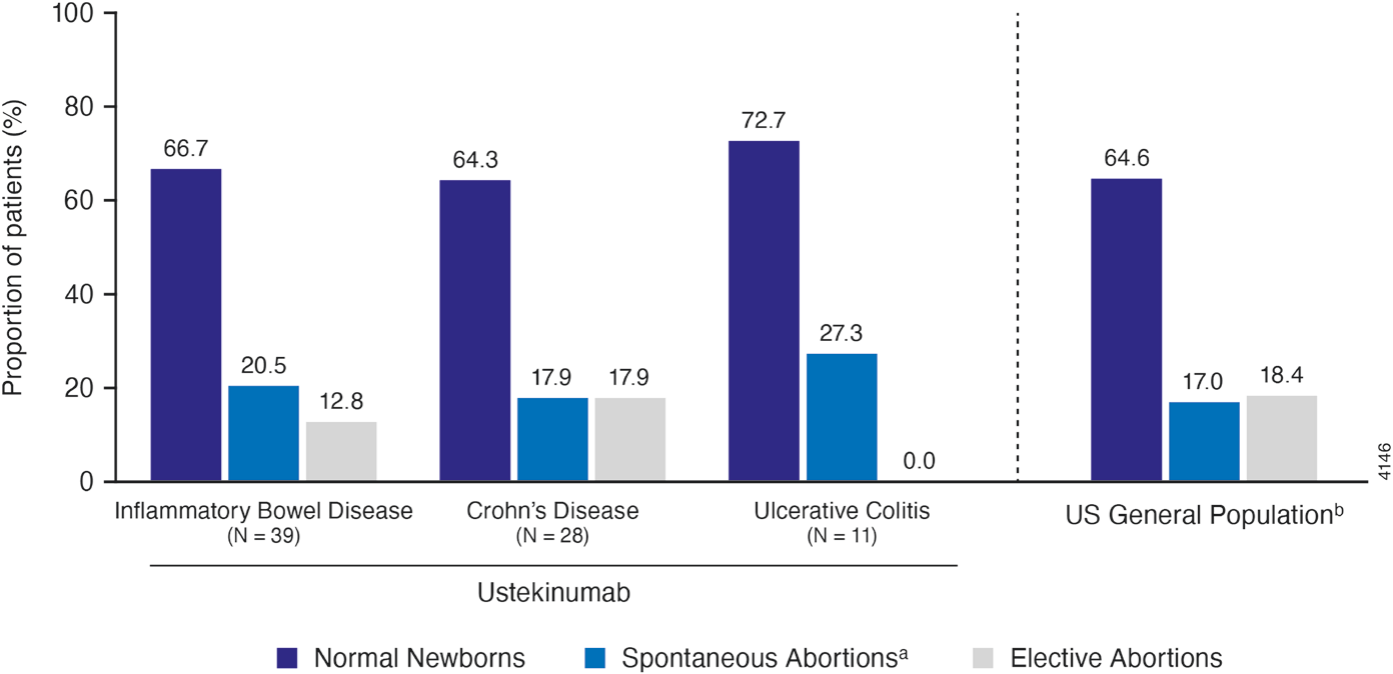
Pregnancy outcomes compared with the US general population. ^a^All spontaneous abortions occurred during the first trimester. ^b^Ventura *et al.*^29^

Pregnancy outcomes across the IBD population included 66.7% (26/39) live births without congenital anomalies, 20.5% (8/39) spontaneous abortions, and 12.8% (5/39) elective abortions (Figure 2). There were no congenital anomalies or stillbirths reported in the IBD pregnancy cohort. Overall, these reported rates are generally comparable to those observed in the general US population (Figure 2).^29^

**Figure 3.**
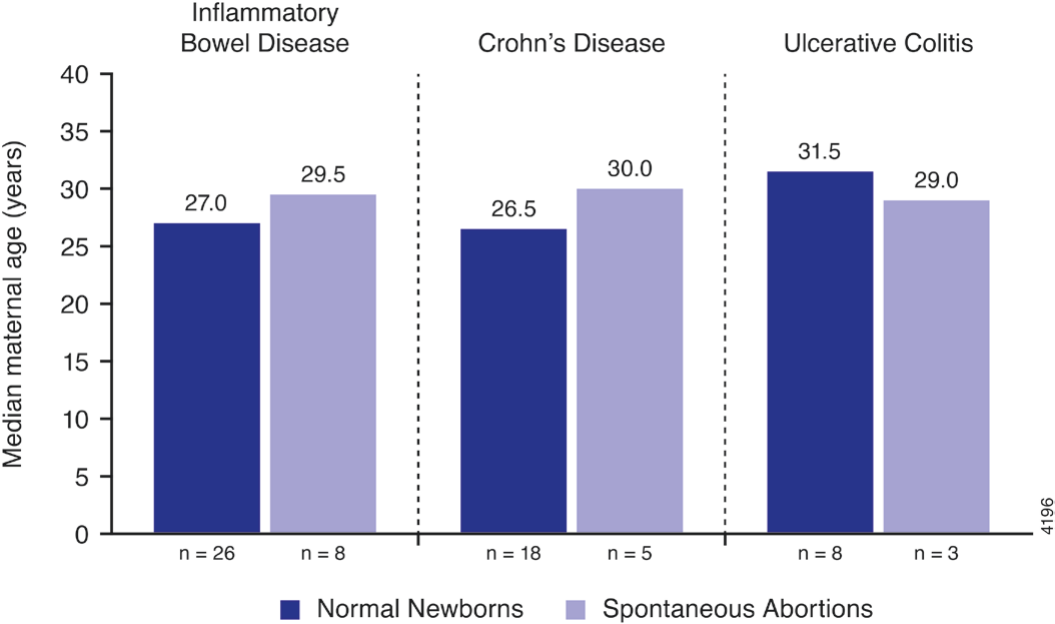
Maternal age by pregnancy outcome.

Median maternal age by pregnancy outcome is presented in Figure 3. In the IBD pregnancy cohort, maternal ages were generally similar between normal newborn outcomes and spontaneous abortions. Women with CD who had a spontaneous abortion were older than those who had a normal newborn outcome (30 and 26.5 years, respectively).

#### Live Births

Of 26 women who had a live birth, all resulted in normal newborns with no congenital anomalies reported. The only AE reported in a live birth was 1 case of hypoglycemia. The median maternal age of these 26 women was 27.0 years, with a median duration of disease of 7.1 years (Table 2). Almost 50% (12/26) were prior TNF failures and approximately a quarter were current smokers (7/26; 26.9%).

Of the 26 normal newborns in the IBD pregnancy cohort, characteristics are available for 22 (15 from CD studies and 7 from the UC study; Table 3). Overall, in the IBD cohort, median gestational age was consistent with term birth (preterm is considered to be <37 weeks^30^). In addition, median birth weight for the IBD pregnancy cohort was 7.1 lbs (low birth weight is considered to be <2500 g^31^ or 5.8 lbs). In the CD pregnancy cohort, the median gestational age of infants born was 38.3 weeks with a median weight of 7.0 lbs. Similar results were seen in the UC pregnancy cohort, with the median gestational age of infants born was 39.3 weeks with a median weight of 7.1 lbs.

**Table 3.** Neonatal outcomes and characteristics.

Health indicator	Inflammatory bowel disease	Crohn’s disease	Ulcerative colitis
Newborn gestational age (weeks)^a^			
*N*	22	15	7
Median (range)	38.4 (33.1, 40.1)	38.3 (36.0, 40.0)	39.3 (33.1, 40.1)
Apgar scores, median (range)^b^			
*N*	12	8	4
Apgar 1-minute score	9.0 (8.0, 10.0)	9.5 (8.0, 10.0)	8.5 (8.0, 9.0)
Apgar 5-minute score	10.0 (8.0, 10.0)	10.0 (8.0, 10.0)	9.0 (8.0, 10.0)
Birth weight (lbs)^c^			
*N*	23	15	8
Median (range)	7.1 (5.3, 9.2)	7.0 (5.3, 8.5)	7.1 (6.2, 9.2)

Abbreviations: lbs, pounds.

Full term is considered to be >37 weeks.

Apgar range is 0–10.

Underweight is considered to be <5 lbs.

Across the IBD cohort, 3 of 22 infants (all in the CD cohort) with reported birth weight were reported as having a low birth weight; though all 3 were >5 lbs. Two of the 3 mothers with low birth weight infants were in remission (CDAI of <150) based on the last CDAI score prior to pregnancy and 1 mother was in response (reduction in CDAI score ≥100 points), but not in remission (decrease in CDAI of 136).

For 12 of 22 infants in the IBD pregnancy cohort, an Apgar score was reported. The median (range) 1-minute Apgar score was 9.0 (8.0, 10.0; scores of 7–10 considered reassuring^32^) and 5-minute score was 10.0 (8–10; Table 3).

#### Spontaneous Abortions

Of the 8 spontaneous abortions reported, 5 were in CD patients and 3 were in UC patients. Additional data on the spontaneous abortions are included in Tables 2 and 4. All spontaneous abortions occurred during the first trimester. Two (25%) of the 8 patients who had a spontaneous abortion were smokers at baseline and 6 (75%) patients had a history of TNF failure. The median age of patients reporting a spontaneous abortion was 29.5 years, and median duration of ustekinumab treatment was 76.4 weeks.

**Table 4.** Characteristics of spontaneous abortions by disease.

Maternal age	Maintenance treatment dosage	Treatment duration (weeks)	Active smoker at baseline?	TNF antagonist failure?	Baseline CDAI/partial Mayo score^a^	Last CDAI/partial Mayo score before pregnancy^a^
Crohn’s disease						
33	90 mg q8w	212	No	Yes	274	54
29	90 mg q8w	57	Yes	Yes	233	63
30	90 mg q8w	115	No	Yes	276	94
31	90 mg q12w	56	Yes	Yes	377	16
28	90 mg q8w	111	No	No	392	84
Ulcerative colitis						
29	90 mg q8w	48	No	Yes^b^	6	3
33	90 mg q8w	96	No	Yes^b^	3	1
23	90 mg q8w	17	No	No	7	1

Abbreviations: q8w, every 8 weeks; q12w, every 12 weeks; TNF, tumor necrosis factor.

Patients with Crohn’s Disease Activity Score (CDAI) <150 or partial Mayo score of 0 or 1 are considered to be in clinical remission.

Patients had also failed vedolizumab treatment.

#### Elective Abortions

All 5 mothers with an outcome of elective abortion had a history of TNF failure. Maternal characteristics are provided in Table 2.

### Maternal Disease Characteristics by Pregnancy Outcomes

**Figure 4.**
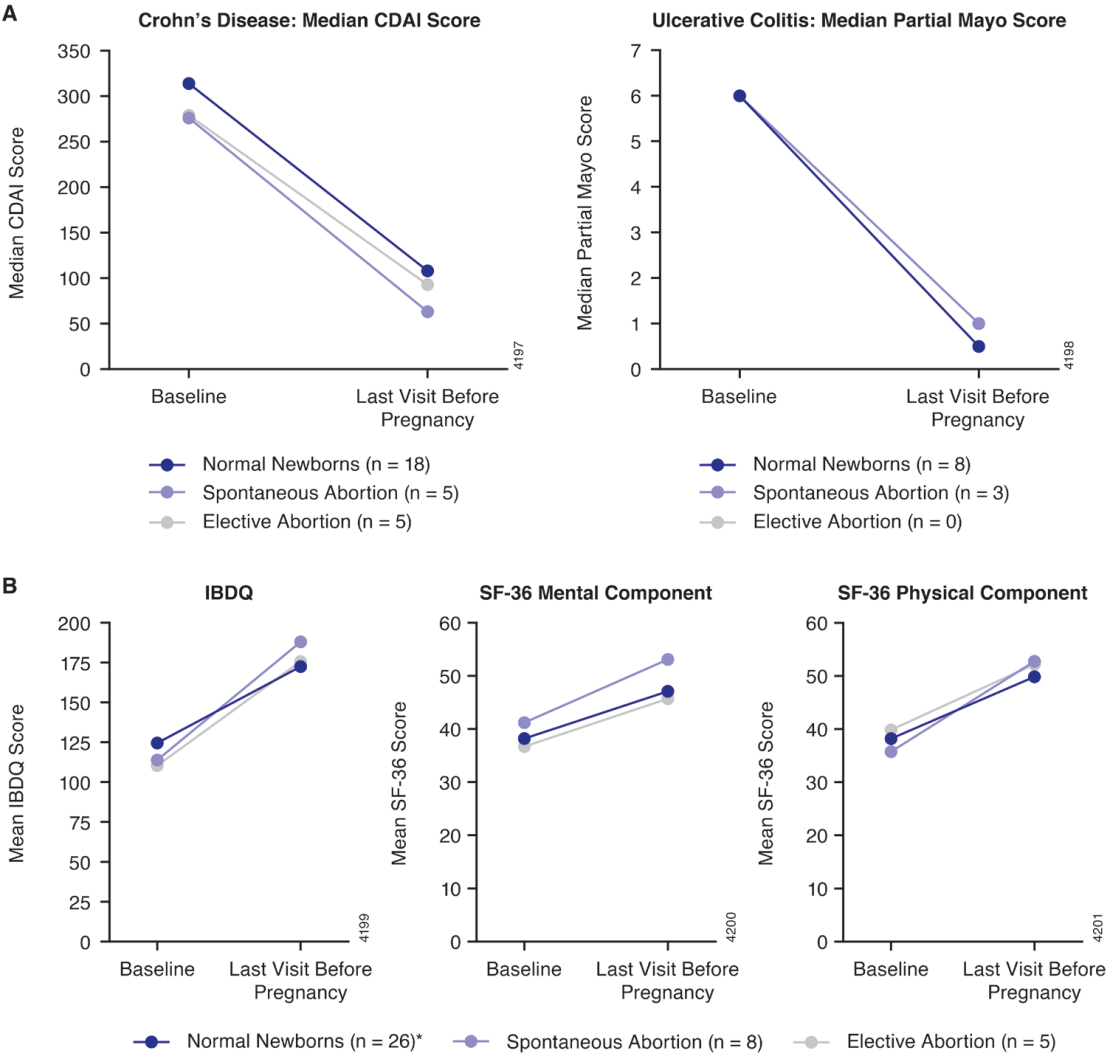
Disease activity (A) and quality of life (B) for CD and UC patients. *n = 24 for SF-36 mental and physical components. Abbreviations: CD, Crohn’s disease; CDAI, Crohn’s Disease Activity Index; IBDQ, Inflammatory Bowel Disease Questionnaire; SF-36, 36-Item Short Form Health Survey; UC, ulcerative colitis.

Baseline CDAI scores and partial Mayo scores were similar for patients with normal newborns, elective abortions, and spontaneous abortions and were representative of a population with moderate-to-severe CD and UC (Figure 4A). The median CDAI score at the visit prior to pregnancy was 88.5 (*n* = 28) and ranged from −11 to 321 (clinical remission defined as CDAI <150^24^). Median partial Mayo score at the visit prior to pregnancy was 1.0 (*n* = 11) and ranged from 1 to 4 (clinical remission defined as partial Mayo score 0 or 1).^26^

Baseline mean IBDQ, and SF-36 scores, for both the mental and physical component summary, and pre-pregnancy and post-baseline scores were generally similar between pregnancy outcomes with clinically meaningful improvements (for IBDQ an increase of ≥16 points and for SF-36 an increase of ≥5 points from baseline is considered clinically meaningful) observed from baseline to pre-pregnancy (IBDQ: 55.7 point improvement; SF-36, mental component: 9.6 point improvement; SF-36, physical component: 12.9 point improvement). Overall, there were no trends between pregnancy outcomes and assessment scores at baseline and prior to pregnancy (Figure 4B).

## Discussion

In this IBD cohort of 39 pregnancies, no specific safety concerns were identified with maternal ustekinumab exposure during pregnancy. No congenital anomalies were reported in newborn infants. Although a small sample, infant characteristics at birth including gestational age, birth weight, and Apgar scores did not identify any safety concerns. Overall, in this pregnancy cohort there does not appear to be an association between pregnancy outcomes and ustekinumab exposure, however in these patients the last dose of ustekinumab was administered prior to or during the first trimester (ustekinumab terminal half-life of ~3 weeks).

Additionally, no increase was observed in the rate of spontaneous abortions (20.5%) in patients treated with ustekinumab compared with the rate reported for the general US population (17%).^29^ Rates of spontaneous abortions reported in this analysis are also generally comparable to rates reported for other biologic treatments prescribed to treat immune-mediated diseases.^20^ Published literature suggest that women with IBD have higher rates of spontaneous abortion than women without IBD.^33^

Data from other pregnancy cohorts, comparing birth outcomes among IBD patients treated with vedolizumab, TNF inhibitors, and biological therapy, have shown that excluding patients with active disease, the risks of adverse outcomes, congenital abnormalities, and number of miscarriages were similar among all treatment groups.^34^ Results from the PIANO registry^19^ suggest biologic treatment during pregnancy in women with IBD was not found to be associated with increased maternal or newborn AEs, consistent with the findings in this IBD pregnancy cohort. Recently, published results for ustekinumab from an observational study, Pregnancy in Crohn’s and Colitis: Levels and Outcomes (PICCOLO), suggest that ustekinumab drug levels are stable during pregnancy in women treated through pregnancy and no new safety signals were identified,^35^ similar to the outcome results reported in PIANO and this pregnancy cohort.

There have also been conflicting suggestions regarding medical treatment during pregnancy. The American Gastroenterological Association clinical care pathway for pregnancy recommends continuing treatment with biologics through all trimesters of the pregnancy for the health of both the mother and child.^18^ As stated in the publication by Mao and Mahadevan,^36^ it is very difficult to differentiate between effects of disease severity and biologic exposure. Risks of uncontrolled IBD and disease flares during pregnancy include preterm deliveries, intrauterine growth restriction, and low birth weight, suggesting that the IBD process may produce greater fetal risk during pregnancy,^19,37,38^ and therefore the risks and benefits of treatments must be considered. As this cohort of pregnant women originated from clinical trials, no patients were actively dosed with ustekinumab once pregnancy was verified as all patients receiving ustekinumab as part of a study were required to discontinue treatment upon pregnancy confirmation (per study protocol), however we have characterized disease activity in these patients based on data from the last visit before pregnancy.

Median CD and UC disease activity measures from study baseline through to the last available assessment before pregnancy, including post-treatment mean CDAI/partial Mayo scores, which were generally comparable across all pregnancy outcome groups, indicated patients were generally doing well with median scores representative of patients in remission, consistent with the overall results reported from the clinical trials.^9–12^ In addition, mean quality of life measure scores, IBDQ and SF-36, indicated clinically meaningful improvements observed from baseline to pre-pregnancy. This reduced level of disease activity may have played a role in the small number of negative pregnancy outcomes (ie, spontaneous abortions and no congenital anomalies) observed.

There are a few limitations to this report including the relatively small sample size of the pregnancy cohort and the mandatory discontinuation of treatment upon pregnancy identification. Overall, the amount of follow-up data on infants born to mothers in this study is limited and the numbers of reported outcomes were small.

Although the results from this analysis represent a small cohort, the results are generally comparable to that reported in children born to mothers with autoimmune diseases receiving TNF antagonists, vedolizumab,^5,39^ or ustekinumab^40^ treatment during pregnancy. In order to fulfill the ongoing need for data, additional information on the impact of ustekinumab in pregnant women is also being studied in the OTIS registry (NCT01086059).

## Conclusion

Based on this series of 39 patients from IBD clinical trials, mothers treated with ustekinumab (limited to the last dose administered up to the first trimester) did not demonstrate a risk of negative outcomes. More data are needed to further characterize the safety profile of ustekinumab use during pregnancy.

## Data Availability

The data sharing policy of Janssen Pharmaceutical Companies of Johnson & Johnson is available at https://www.janssen.com/clinical-trials/transparency. As noted on this site, requests for access to the study data can be submitted through Yale Open Data Access (YODA) Project site at http://yoda.yale.edu.
